# An in vitro investigation into the impact of corneal rinsing on riboflavin/UVA corneal cross-linking

**DOI:** 10.1186/s40662-024-00375-4

**Published:** 2024-02-28

**Authors:** Siân R. Morgan, David P. S. O’Brart, Jinhai Huang, Keith M. Meek, Sally Hayes

**Affiliations:** 1 School of Optometry and Vision Sciences, Cardiff University, Maindy Road, Cardiff, UK; 2 Department of Ophthalmology, Guys and St. Thomas’ NHS Foundation Trust, London, UK; 3 King’s College, London, UK; 4 Eye Institute and Department of Ophthalmology, Eye and ENT Hospital, Fudan University, Key Laboratory of Myopia, Chinese Academy of Medical Sciences, Shanghai, 200030 China

**Keywords:** Cornea, Keratoconus, Corneal cross-linking, Collagen, Collagenase, Enzyme

## Abstract

**Background:**

Corneal cross-linking (CXL) using riboflavin and ultraviolet-A light (UVA) is a treatment used to prevent progression of keratoconus. This ex vivo study assesses the impact on CXL effectiveness, as measured by tissue enzymatic resistance and confocal microscopy, of including a pre-UVA corneal surface rinse with balanced salt solution (BSS) as part of the epithelium-off treatment protocol.

**Methods:**

Sixty-eight porcine eyes, after epithelial debridement, were assigned to six groups in three experimental runs. Group 1 remained untreated. Groups 2–6 received a 16-min application of 0.1% riboflavin/Hydroxypropyl methylcellulose (HPMC) drops, after which Group 3 was exposed to 9 mW/cm^2^ UVA for 10 min, and Groups 4–6 underwent corneal surface rinsing with 0.25 mL, 1 mL or 10 mL BSS followed by 9 mW/cm^2^ UVA exposure for 10 min. Central corneal thickness (CCT) was recorded at each stage. Central 8.0 mm corneal buttons from all eyes were subjected to 0.3% collagenase digestion at 37 °C and the time required for complete digestion determined. A further 15 eyes underwent fluorescence confocal microscopy to assess the impact of rinsing on stromal riboflavin concentration.

**Results:**

Application of riboflavin/HPMC solution led to an increase in CCT of 73 ± 14 µm (*P* < 0.01) after 16 min. All CXL-treated corneas displayed a 2–4 fold greater resistance to collagenase digestion than non-irradiated corneas. There was no difference in resistance between corneas that received no BSS rinse and those that received a 0.25 mL or 1 mL pre-UVA rinse, but each showed a greater level of resistance than those that received a 10 mL pre-UVA rinse (*P* < 0.05). Confocal microscopy demonstrated reduced stromal riboflavin fluorescence after rinsing.

**Conclusions:**

All protocols, with and without rinsing, were effective at enhancing the resistance to collagenase digestion, although resistance was significantly decreased, and stromal riboflavin fluorescence reduced with a 10 mL rinse. This suggests that a 10 mL surface rinse can reduce the efficacy of CXL through the dilution of the stromal riboflavin concentration.

## Background

Keratoconus is a sight-limiting condition characterised by ectatic bulging of the cornea [1]. This process of pathological degeneration is associated with an upregulation of degradative enzymes [2] and abnormalities in the orientation and distribution of collagen within the corneal stroma [3].

In 2003, Wollensak et al. first demonstrated the clinical potential of riboflavin/ultraviolet-A light (UVA) corneal cross-linking (CXL) for the stabilisation of progressive keratoconus [4]. Since then, the effectiveness of corneal cross-linking as a means of safely and effectively halting keratoconus progression has been proven in numerous clinical trials [5–7]; this has resulted in the treatment gaining worldwide acceptance. The original CXL protocol described by Wollensak et al. [4] involved removal of the corneal epithelium followed by a 30-min application of a 0.1% riboflavin, in 20% dextran, and a 30-min exposure to UVA irradiation at 370 nm and 3 mW/cm^2^. Although this protocol, referred to as the ‘Dresden protocol’, is still in use, many variations have been developed to reduce patient discomfort and treatment time. Such variations include accelerated CXL protocols which make use of higher UVA intensities to deliver the same energy dose over a shorter period [8], with 10-min UVA exposure to 9 mW/cm^2^ or 5-min exposure to 18 mW/cm^2^, having been shown to be clinically effective [9–11]. There has also been a shift away from the use of hypertonic riboflavin solutions containing dextran, which can induce corneal thinning from dehydration, towards the use of isotonic riboflavin solutions containing hydroxypropyl methylcellulose (HPMC) [10]. These solutions offer an enhanced rate of diffusion into the corneal stroma [12] with reduced effects on corneal thickness [13, 14]. Differences in the physio-chemical properties of the dextran and HPMC carrier solutions result in the riboflavin film covering the corneal surface being significantly thicker with riboflavin/HPMC solutions and the film break-up time being longer [15]. As a result, some ophthalmologists have chosen to routinely rinse the cornea prior to UVA exposure, with the aim of removing the superficial riboflavin film to minimise UVA absorption at the corneal surface and maximise absorption within the stroma [10]. However, laboratory studies examining transepithelial riboflavin penetration have shown that excessive corneal rinsing can lead to the dilution of riboflavin from the anterior stroma, limiting riboflavin availability for photodynamic cross-linking within this important structural region [16]. It can be postulated that this potential for riboflavin washout from the anterior stroma might be even greater when rinsing is performed during epithelium-off CXL.

To date, there is a paucity of published scientific studies examining the effect of surface rinsing procedures on cross-linking efficiency. To address this, we examined the impact of rinsing riboflavin, containing HPMC, from the surface of the de-epithelialised cornea prior to UVA exposure, using clinically relevant (0.25 to 1 mL) and above (10 mL) volumes of balanced salt solution (BSS). To assess CXL effectiveness, confocal microscopy was used to evaluate stromal riboflavin penetration and corneal enzymatic resistance, which are known to be enhanced after CXL [17, 18] (due to changes in the tertiary structure of collagen hindering proteolytic cleavage via steric hindrance).

## Methods

### Enzymatic digestion studies

Due to sample availability and the desire to treat all corneas within 12 h of death to minimise the effects of posthumous corneal swelling, this study was conducted over the course of three separate experimental runs. Run 1 comprised a preliminary study to determine the impact of riboflavin treatment (without UVA exposure) on corneal enzymatic resistance and Runs 2 and 3 assessed CXL effectiveness in corneas treated with and without a pre-UVA rinse and ensured reproducibility of the most clinically relevant findings.

In total, 68 porcine eyes with clear, intact corneas were received on ice from a licenced European abattoir within 6 h of death. The eyes were returned to room temperature immediately prior to use and divided into the six groups detailed below. In all eyes, the corneal epithelium was removed using a single edged razor blade.

Group 1: Untreated control (Run 1: n = 7; Run 2: n = 10; Run 3: n = 6): De-epithelialised cornea that received no riboflavin or UVA irradiation.

Group 2: Riboflavin only control (Run 1: n = 5): 0.1% riboflavin in 1.0% HPMC (Mediocross M, Avedro Inc., USA) was applied to the de-epithelialised corneal surface at 2-min intervals for 16 min.

Group 3: Cross-linking (CXL) with no BSS rinse (Run 2: n = 5; Run 3: n = 10): 0.1% riboflavin in 1.0% HPMC was applied to the de-epithelialised corneal surface at 2-min intervals for a total of 16 min. This was immediately followed by a 10-min exposure to 365 nm UVA light with an irradiance of 9 mW/cm^2^ using a CCL-Vario cross-linking device (Peschke M, Peschke trade GmbH, Huenenberg, Switzerland) set to a beam diameter of 11 mm.

Group 4: CXL with a 0.25 mL pre-UVA BSS rinse (Run 2: n = 5; Run 3: n = 10): 0.1% riboflavin in 1.0% HPMC was applied to the de-epithelialised corneal surface at 2-min intervals for a total of 16 min. This was followed by a rinse of the corneal surface with 0.25 mL BSS (Dulbecco’s Phosphate buffered saline), and a 10-min exposure to 365 nm UVA light with a fluence of 9 mW/cm^2^.

Group 5: CXL with a 1 mL pre-UVA BSS rinse (Run 2: n = 5): 0.1% riboflavin in 1.0% HPMC was applied to the de-epithelialised corneal surface at 2-min intervals for a total of 16 min. This was followed by a rinse of the corneal surface with 1 mL BSS (Dulbecco’s Phosphate buffered saline), and a 10-min exposure to 365 nm UVA light with a fluence of 9 mW/cm^2^.

Group 6: CXL with a 10 mL pre-UVA BSS rinse (Run 2: n = 5): 0.1% riboflavin in 1.0% HPMC was applied to the de-epithelialised corneal surface at 2-min intervals for a total of 16 min. This was followed by a rinse of the corneal surface with 10 mL BSS, and a 10-min exposure to 365 nm UVA light with a fluence of 9 mW/cm^2^.

In each experimental run, one eye from each group was treated in sequence to ensure that posthumous corneal swelling effects were evenly distributed amongst the groups. The riboflavin instillation time was increased from the manufacturer recommended 10 to 16 min to account for the greater thickness of porcine corneas compared to human corneas and ensure maximal riboflavin penetration throughout the thicker porcine corneal stroma. For corneas undergoing a surface rinse as part of their treatment (Groups 4–6), BSS was used to avoid any potential changes in stromal pH, which might adversely affect oxygen availability and hinder the cross-linking process [19], and this was applied in a dropwise manner (at a rate of approx. 0.5 mL/s). The central corneal thickness (CCT) of each eye was measured using a SP-100 portable pachymeter (Tomey GmbH Technology and Vision, Nurnberg, Germany) before and after epithelium debridement, and following each stage of treatment (post-riboflavin application, post-rinse, and post-UVA exposure, where applicable). Immediately after completion of each treatment, the surface of the cornea was gently wiped with a tissue to remove any excess riboflavin/BSS and an 8.0-mm full-tissue thickness disk was trephined from the centre of each cornea. The corneal disks were then wrapped tightly in catering film and refrigerated until all corneas had been treated, after which they were returned to room temperature.

Corneal disks were placed into individual wells each containing a 2 mL solution of 0.3% Collagenase A obtained from *Clostridium histolyticum* (Sigma, UK) and incubated at 37 °C. In Runs 1 and 2, samples were maintained in a static incubator. In Run 3, to ensure continuous sample agitation and expedite the rate of digestion, a 37 °C/200 rpm incubator-shaker (Incu-Shake MIDI, SciQuip, Newtown, United Kingdom), was used. At regular intervals (every 1.5 to 2 h during the day and at 8-hourly intervals during the night) the samples were removed from their respective incubators and examined under a microscope to assess their integrity. In each run, the time required for total tissue digestion was recorded for each sample.

### Confocal microscopy studies

#### Sample preparation

A further 15 porcine eyes with clear, intact corneas were obtained on ice from the abattoir within 6 h of death and divided into five groups. The corneas underwent the same 16-min riboflavin application and corneal rinse protocols described previously for Groups 1, 3 to 6 (n = 3 per group) but importantly, these samples remained unirradiated. Post treatment, the corneas were immediately excised, immersed in liquid nitrogen for 5 min and transferred to storage at − 80 °C. A 1-mm wide, full-tissue thickness corneal strip was cut from the center of each cornea (along the horizontal meridian), and the cross-sectional surface of each strip was placed face-down onto a microscope slide. A CoverWell™ Imaging Chamber Gasket (Thermofisher, UK) was placed on top of the sample. To minimise the migration of riboflavin within the corneal strip prior to imaging, no immersion fluid was used. The time taken to excise and mount the tissue prior to imaging was under 2 min.

#### Confocal microscope setup and imaging protocol

Riboflavin penetration was evaluated by measuring the fluorescence intensity of riboflavin within the anterior-most region of the stroma. Samples were imaged on a Zeiss LSM 880 META NLO microscope (Carl Zeiss Ltd, Welwyn Garden City, UK). Based on the absorption spectrum for riboflavin, an excitation wavelength of 458 nm was used. A single image (using a 20X/0.8 NA air objective) was obtained at a resolution of 1024 by 1024 pixels per image and with a lateral resolution of 0.42 μm per pixel. Experimental settings (including detector gain) were unchanged for all samples to ensure comparable intensity values.

### Imaging analysis

Grayscale images were exported and analysed using ImageJ imaging software [20]. The plot profile tool in ImageJ was used to calculate the fluorescence intensity of riboflavin as a function of tissue depth. A line profile was drawn normal to the anterior-most surface of the cornea down to a depth of 300 µm, and the fluorescence intensity profiles for each image were exported into Excel. The fluorescence intensity data for each image was then averaged within each treatment group (n = 3 images per treatment group) and plotted as intensity profiles to show the fluorescence intensity of riboflavin as a function of tissue depth.

### Statistical analysis

Changes in CCT between the different stages of treatment were statistically assessed by means of paired t-tests. Comparisons between groups in terms of the time required for total digestion were performed using single-factor ANOVA and post hoc least significant difference testing. Data are shown as mean measurements (± SD) for corneal thickness and digestion times. All statistical analyses were performed using Microsoft Excel. A probability value of less than 0.05 was considered significant.

## Results

### Corneal thickness

**Fig. 1:**
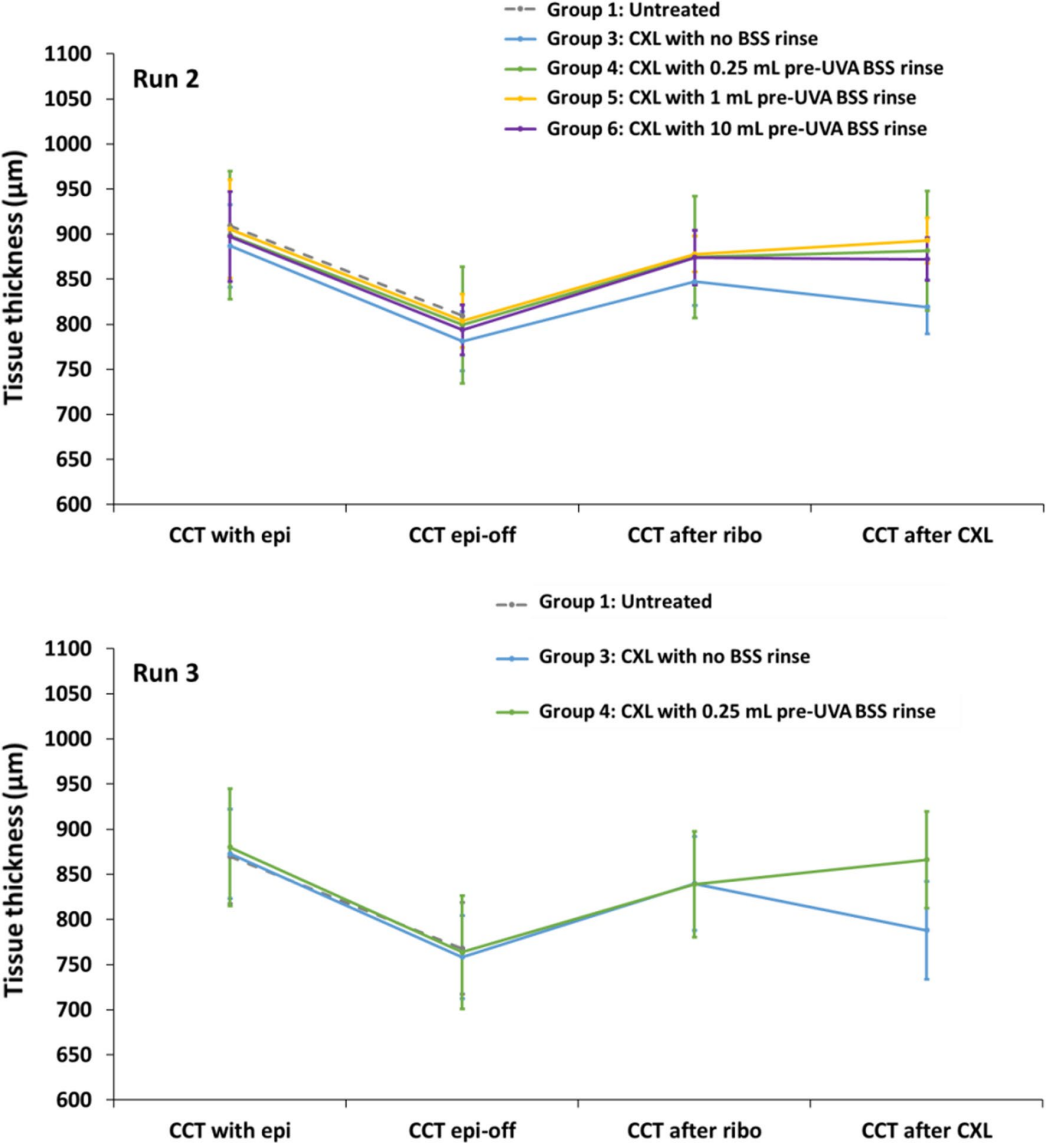
Average central corneal thickness (CCT) for each treatment group in Run 2 (top) and Run 3 (bottom) as measured before (CCT with epi) and after (CCT epi-off) removal of the epithelium, following riboflavin application (CCT after ribo) and following the completion of the cross-linking treatment (CCT after CXL). CXL, corneal cross-linking; UVA, ultraviolet-A light; BSS, balanced salt solution

Measurements of CCT before and after removal of the epithelium, following riboflavin application and completion of each cross-linking treatment are shown in Fig. 1. In Runs 2 and 3, the differences between groups in terms of corneal thickness before and after removal of the epithelium were not significant. Application of riboflavin/HPMC to the de-epithelialised cornea resulted in a significant increase in CCT of 73 ± 14 µm (*P* < 0.01) after 16 min, which amounts to a 9% increase in stromal thickness. There was no significant difference in final CCT measurements of the untreated and unrinsed CXL groups. In all cases, the CCT measurements for the 0.25 mL (Group 4), 1 mL (Group 5) and 10 mL (Group 6) rinse CXL treatments were unchanged immediately after rinsing (data not shown) but following a 10-min UVA exposure, they became significantly higher than that of the de-epithelialised untreated corneas (Group 1) (*P* < 0.01).

### Time taken for complete digestion

Our preliminary study (Run 1) showed no significant difference between untreated (Group 1), and riboflavin only treated (Group 2) corneas in the time taken to reach complete digestion (data not shown), justifying the subsequent use of a single control group (untreated corneas) in Runs 2 and 3.

**Fig. 2:**
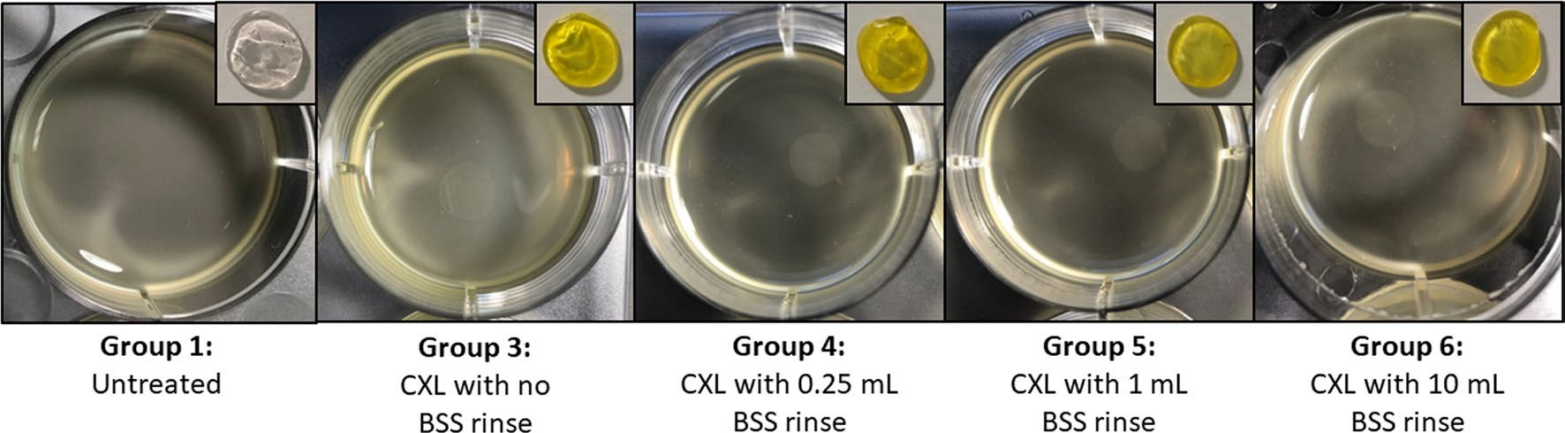
Corneal disks from Run 2 before (inset) and after collagenase digestion. After 33 h in 0.3% collagenase solution, most untreated samples (Group 1) were fully digested, but all CXL treated corneal disks (Groups 3–6) remained intact, showing enhanced resistance to enzymatic digestion. CXL, corneal cross-linking; BSS, balanced salt solution

**Fig. 3:**
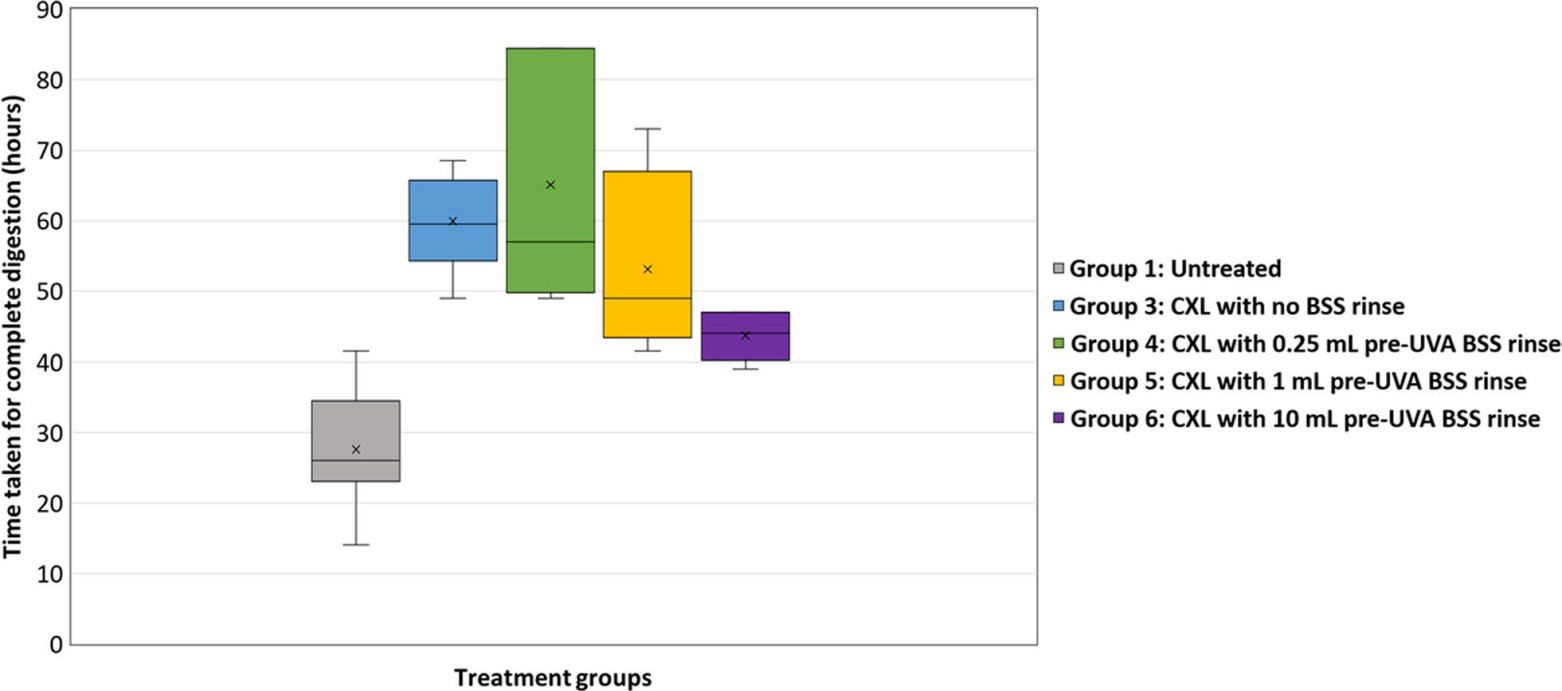
Average time taken for complete digestion of untreated and cross-linked (CXL) corneas in Run 2. CXL treated corneas (Groups 3–6) showed enhanced resistance to enzymatic digestion compared to untreated corneas (*P* < 0.01 for Groups 3–5;* P* < 0.05 for group 6). CXL, corneal cross-linking; UVA, ultraviolet-A light; BSS, balanced salt solution

**Fig. 4:**
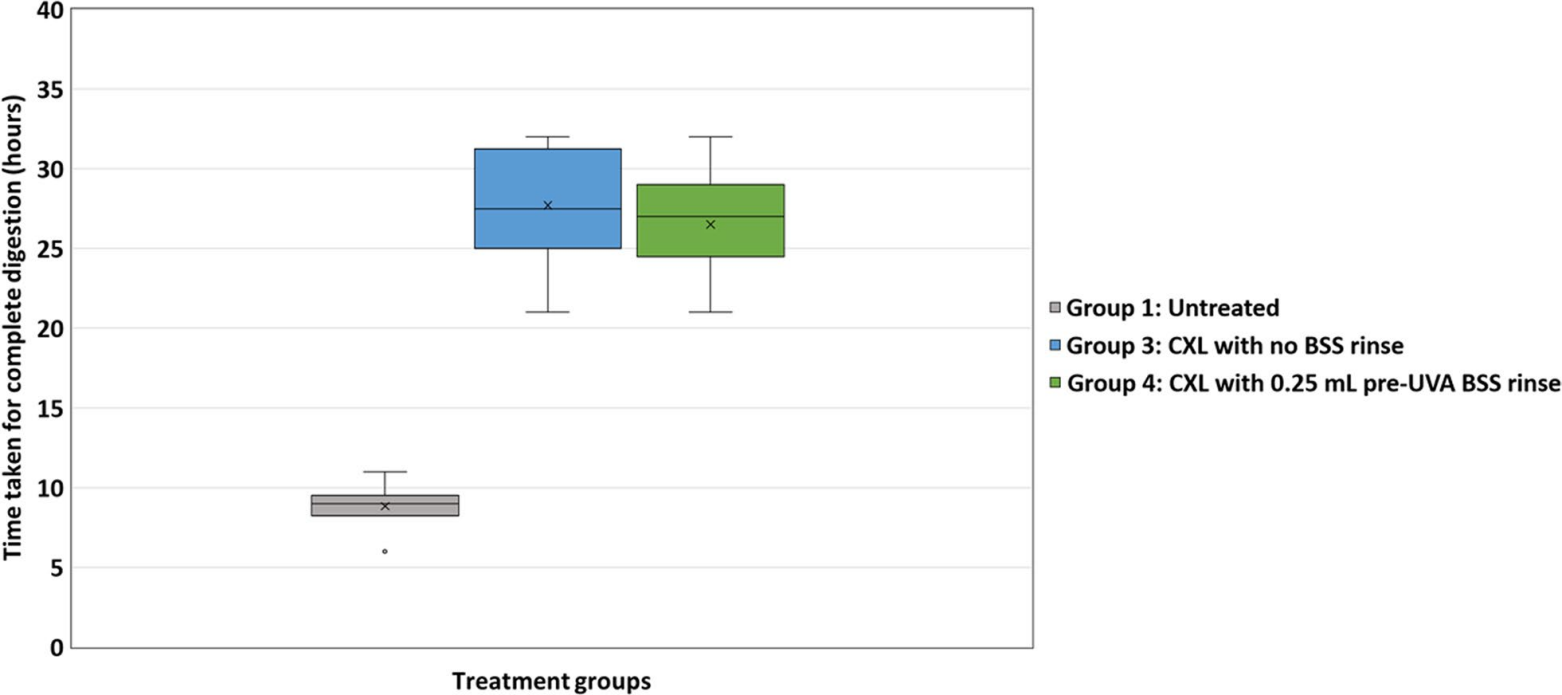
Average time taken for complete digestion of untreated and cross-linked (CXL) corneas in Run 3. CXL treated corneas (Groups 3 and 4) showed enhanced resistance to enzymatic digestion compared to untreated corneas (*P* < 0.01). CXL, corneal cross-linking; UVA, ultraviolet-A light; BSS, balanced salt solution

In Runs 2 and 3 (Figs. 2, 3 and 4), CXL treated corneas (Groups 3–6) showed enhanced resistance to enzymatic digestion compared to untreated corneas (Run 2: *P* < 0.01 for Groups 3–5; *P* < 0.05 for group 6; Run 3: *P* < 0.01 for Groups 3 and 4). As shown in both experimental Runs 2 and 3, there was no difference in the enzymatic resistance of corneas that were cross-linked without a rinse (Group 3) and those that received a 0.25 mL pre-UVA rinse (Group 4). However, data from Run 2 revealed a trend for the enzymatic resistance of cross-linked corneas to decrease as the rinse volume increased above 0.25 mL. Although the difference in the time required for complete digestion of the corneas that received a 0.25 mL pre-UVA rinse (Group 4) and those that received a 1 mL rinse (Group 5) failed to reach significance compared to non-rinsed corneas, the enzymatic resistance of the 10 mL rinse group (Group 6) was significantly less than that of all other cross-linked groups (Groups 3–5) (*P* < 0.01 for Group 4; *P* < 0.05 for Groups 3 and 5) (Fig. 3).

### Confocal microscopy

**Fig. 5:**
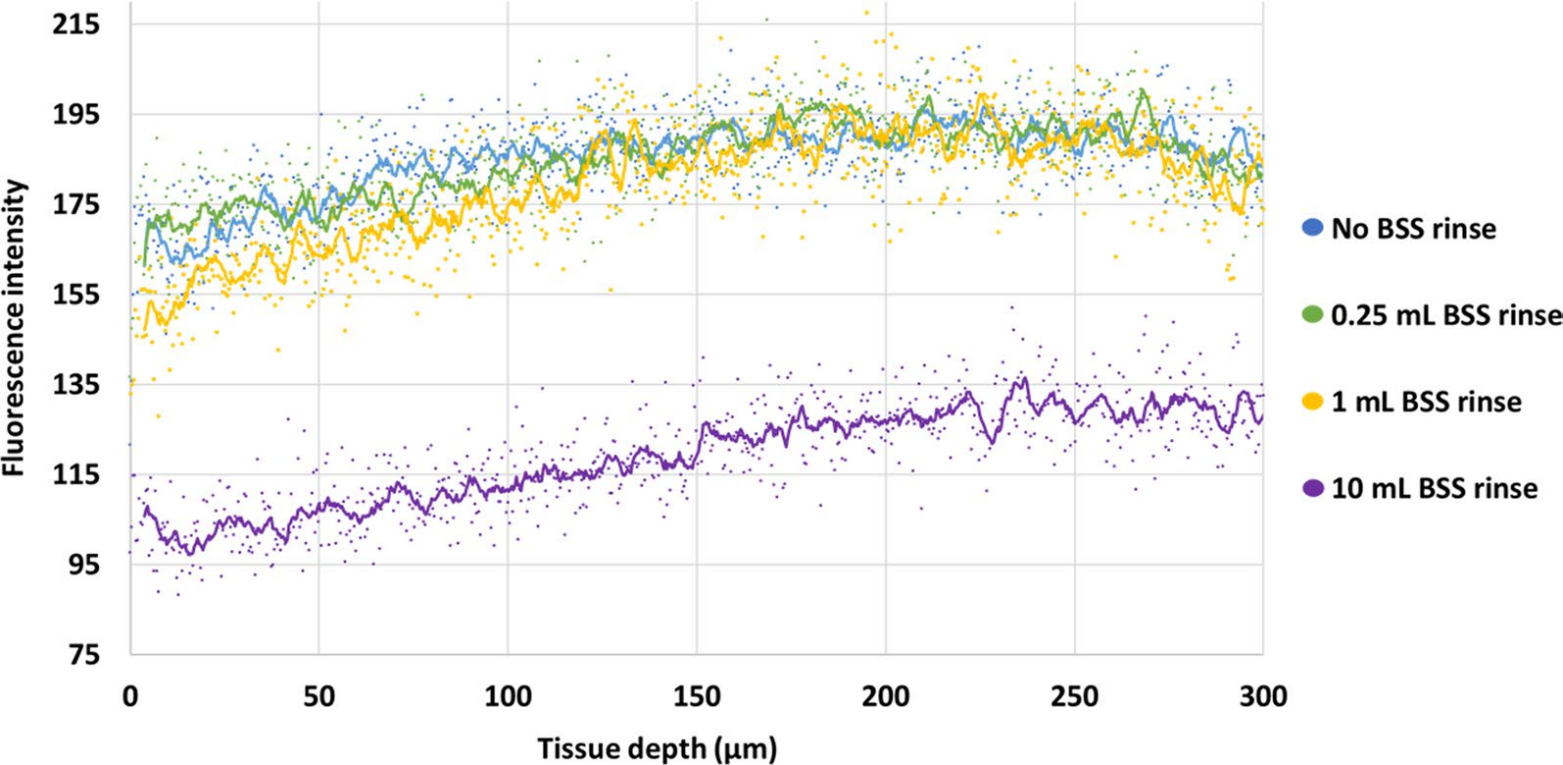
Graphical representation of fluorescence intensity profiles through the anterior-most region of the corneal stroma (0 to 300 μm) following application of a 0.1% riboflavin/1.0% HPMC solution with no BSS rinse (Group 3), a 0.25 mL rinse (Group 4), 1 mL rinse (Group 5) or 10 mL rinse (Group 6) (n = 3 per group). The 10 mL BSS rinse group showed a significantly lower intensity in the anterior-most 300 µm compared to the no rinse (*P* < 0.05), 0.25 mL rinse (*P* < 0.05) and 1 mL rinse (*P* < 0.05) groups. Trendlines: Moving average using 10 data points. CXL, corneal cross-linking; UVA, ultraviolet-A light; BSS, balanced salt solution; HPMC, hydroxypropyl methylcellulose; CCT, central corneal thickness

The fluorescence intensity (which correlates with the concentration of riboflavin in the stroma) was visibly less in the samples that underwent a 10 mL BSS rinse compared with those that had no rinse or were rinsed with a smaller volume of BSS (1 mL or less) (data not shown). When the fluorescence intensity of riboflavin was averaged by treatment group and displayed as a function of tissue depth (Fig. 5), the intensity values of the no rinse, 0.25 mL rinse and 1 mL rinse groups were found to be closely aligned with each other but the high volume (10 mL) BSS rinse group showed a significantly lower intensity in the anterior 300 µm of the corneal stroma compared to the no rinse (*P* < 0.05), 0.25 mL rinse (*P* < 0.05) and 1 mL rinse (*P* < 0.05) groups.

## Discussion

Since the first reported clinical trial of CXL [4], numerous studies have focussed on the development of treatment modifications to reduce treatment time and increase patient comfort, using differing riboflavin solutions [12, 16, 18, 21–23] and changes in the intensity and duration of UVA exposure [9, 17, 24–26]. However, as identified in a recent survey of corneal cross-linking practice in the UK [10], there are other seemingly more minor treatment variations in current use, and the impact of these on CXL effectiveness have yet to be investigated. For example, some ophthalmologists choose to routinely rinse the surface of the cornea prior to UVA exposure with the aim of removing the surface riboflavin film to reduce its possible masking effect and optimise stromal absorption of UVA [10]. Unfortunately, there is insufficient experimental evidence supporting the benefit of this treatment modification and the volume of the rinse remains unstandardised. In this study, the enzymatic resistance of corneas cross-linked with and without a pre-UVA BSS rinse was examined to determine the impact of corneal rinsing on CXL effectiveness and confocal microscopy was used to examine stromal riboflavin penetration.

Ensuring adequate corneal stromal saturation and concentration of riboflavin is vital to ensure optimal CXL [18, 21]. Riboflavin acts as a photosensitising dye to initiate photochemical reactions to create cross-links between the corneal proteins as well as behaving as a shield to protect the corneal endothelium, lens, and retina from UVA damage [27]. The current preferential use of isotonic riboflavin in a HPMC carrier solution by many ophthalmologists [10], in preference to the riboflavin/dextran solution used in the original cross-linking protocol, is a result of its faster diffusion time, reducing the time required for riboflavin application [12]. A further benefit of riboflavin/HPMC solutions, is that unlike riboflavin/dextran solutions, they do not cause significant tissue dehydration [23]. Indeed, clinical studies have shown that the application of riboflavin/HPMC solution has little effect on corneal hydration, producing a small increase [23] or decrease [14] in corneal thickness during treatment. In tandem with the clinical findings of Zaheer et al. [23] and the laboratory studies of Fischinger et al. [28] on porcine corneas, this study revealed a significant increase in CCT (9%) following a 16-min application of riboflavin/HPMC to the de-epithelialised cornea. Furthermore, we showed that the post-treatment thickness of corneas that underwent CXL without a BSS rinse prior to UVA exposure, was similar to that of untreated (epithelium-removed) corneas. However, corneas that received a pre-UVA rinse with BSS of 0.25 mL or more, exhibited a significantly higher post-treatment thickness compared to the untreated corneas. It can be postulated that this increase in thickness is due to stromal hydration directly from the BSS, which when used as a hydration fluid during ocular surgery and eye examination procedures, is known to cause a period of temporary corneal swelling [29].

As riboflavin behaves like a fluorescent chromophore, emitting a greenish-yellow fluorescence, fluorescence microscopy (using either single-photon (i.e., confocal) or multiphoton excitation) can be used to investigate the intensity and distribution of riboflavin throughout the cornea [12, 16, 30]. Using two-photon fluorescence microscopy [12], it has been shown that the stromal riboflavin distribution within the anterior 350 µm of porcine corneas after 10 min of imbibition with a 0.1% riboflavin/HPMC solution is comparable to the riboflavin distribution after 30 min of imbibition with aqueous 0.1% riboflavin/20% dextran solution. However, it should be noted that porcine eyes, which are frequently used in ophthalmic research due to their ready availability and anatomic similarities with the human eye [31], possess a thicker cornea due to the presence of a thicker epithelium and stroma [32]. Here, the variation in corneal epithelium thickness can be disregarded as it was removed from the porcine eyes prior to use, and the difference in stromal thickness was overcome by increasing the riboflavin/HPMC instillation time from 10 to 16 min to ensure stromal saturation of riboflavin throughout the entire cornea.

In theory, removal of the riboflavin film from the outer-most surface of the cornea prior to UVA exposure could increase UVA absorption within the stroma and enhance the cross-linking procedure. This may be especially true in the case of riboflavin/HPMC solutions which produce particularly thick riboflavin films [15]. However, it has been also argued that the presence of a stable surface riboflavin film may help prevent tissue dehydration during CXL [4, 15] and maintain a riboflavin steady-state equilibrium within the corneal stroma [4, 15, 27]. As such, excessive rinsing of the corneal surface could not only lead to the depletion of riboflavin from the surface, but also from the anterior stroma, which would be expected to reduce CXL efficacy [22]. It is therefore not surprising that the reduced availability of stromal riboflavin following an extensive 10 mL BSS rinse (as shown by our confocal microscopy results), led to a reduced cross-linking effect, which was evidenced by a lower enzymatic resistance of these corneas compared to those that received no pre-UVA rinse or a lower volume rinse (Fig. 3). Although the influence of riboflavin film thickness on corneal oxygen permeability was not measured in this study, the absence of any detectable difference in the enzymatic resistance of the unrinsed CXL treated corneas (which had surface riboflavin) and the 0.25 mL rinsed CXL corneas (which had no surface riboflavin or loss of stromal riboflavin) suggests that the presence/absence of surface riboflavin does not significantly affect the CXL process when using a 9mW/10 min protocol. However, the presence of a surface riboflavin film may have a greater effect on corneal CXL when higher UVA intensities are used due to the more rapid depletion of oxygen within the corneal stroma [33].

## Conclusion

The results of this study indicate that extensive rinsing of the cornea with 10 mL of BSS solution to remove riboflavin film from the corneal surface prior to UVA exposure has a negative impact on CXL due to the depletion of riboflavin from the anterior stroma and should therefore be avoided. If surface irrigation is indicated, the volumes of 1 mL or less have fewer detrimental effects on CXL efficacy and anterior stromal riboflavin concentration. Although the inclusion of a low volume surface rinse with BSS prior to UVA exposure does not provide any detectable advantage or disadvantage in terms of CXL effectiveness, it may offer some benefit in terms of tissue hydration, particularly for the treatment of very thin keratoconus corneas (less than the 400 µm), which require tissue swelling to reach the minimum safe thickness threshold for treatment [34]. Further studies are required to assess these modifications to the corneal cross-linking protocol and develop evidence-based guidelines for best practice.

## Data Availability

All the data supporting the findings of this study are available within the article.

## Abbreviations

CXL: Corneal cross-linking
UVA: Ultraviolet-A light
BSS: Balanced salt solution
HPMC: Hydroxypropyl methylcellulose
CCT: Central corneal thickness
